# Alteration of m^6^A epitranscriptomic tagging of ribonucleic acids after spinal cord injury in mice

**DOI:** 10.3389/fnins.2022.904573

**Published:** 2022-08-25

**Authors:** Shuangfei Ni, Zixiang Luo, Yonggang Fan, Weixin Zhang, Wei Peng, Huafeng Zhang

**Affiliations:** ^1^Department of Orthopaedics, The First Affiliated Hospital of Zhengzhou University, Zhengzhou, China; ^2^Department of Spine Surgery and Orthopaedics, Xiangya Hospital, Central South University, Changsha, China; ^3^Department of Orthopaedics, Zhejiang Chinese Medicine University, Hangzhou, China

**Keywords:** spinal cord injured (SCI), m^6^A (N6-methyladenosine), METTL3 (methyltransferase like 3), RNA, microarray

## Abstract

The m^6^A methylation is reported to function in multiple physiological and pathological processes. However, the functional relevance of m^6^A modification to post-spinal cord injured (SCI) damage is not yet clear. In the present study, methylated RNA immunoprecipitation combined with microarray analysis showed that the global RNA m^6^A levels were decreased following SCI. Then, gene ontology (GO) and kyoto encyclopedia of genes and genomes (KEGG) analyses were conducted to demonstrate the potential function of differential m^6^A-tagged transcripts and the altered transcripts with differential m^6^A levels. In addition, we found that the m^6^A “writer,” METTL3, significantly decreased after SCI in mice. The immunostaining validated that the expression of METTL3 mainly changed in GFAP or Iba-1^+^ cells. Together, this study shows the alteration of m^6^A modification following SCI in mice, which might contribute to the pathophysiology of the spinal cord after trauma.

## Introduction

Spinal cord injury (SCI) is a devastating pathological status that results in persistent functional deficits and high mortality (Ahuja et al., 2017). The prevalent cases of SCI were approximately 27.04 million worldwide (GBD 2016 Neurology Collaborators, 2019). Numerous significant advances in medical treatment have been achieved in experimental SCI models, but no definitive therapies exist for SCI in the clinic. The development of an effective treatment strategy is limited by an incomplete understanding of the pathological mechanisms that occur at different stages after SCI. The intricate biological processes and molecular events, namely, excitotoxicity, ionic imbalance, oxidative stress, endoplasmic reticulum stress, apoptosis, and inflammation, govern the neuronal fate and affect neurological functional recovery after SCI (Mehta et al., 2007; Fan et al., 2018). In recent years, RNA modification has been reported to function in these biological processes and molecular events (Wen et al., 2020; Zhang et al., 2020; Wang et al., 2021b; Yang and Chen, 2021; Yu et al., 2021; He et al., 2022).

In the process of epigenetic regulation, RNAs, which are similar to DNA or histone, could undergo over 100 kinds of posttranscriptional modifications in mammals (Cantara et al., 2011; Niu et al., 2013). The internal epi-transcriptomic changes include N1-methyladenosine (m^1^A), N5-methylcytosine (m^5^C), N6-methyladenosine (m^5^A), and pseudouridine (ψ; Wei et al., 2018; Weng et al., 2018). Among them, m^6^A, which can regulate RNA structure, stability, and expression, is regarded as the most universal and reversible modification of all messenger RNA (mRNA) and non-coding RNA base methylations in eukaryotic cells (Roundtree et al., 2017; Zhao et al., 2017; Frye et al., 2018). The latest research shows that m^6^A modification is mediated mainly by various “writer,” “reader,” and “eraser” proteins (Meyer and Jaffrey, 2017), such as methyltransferase-like (METTL) 3 and 14, Wilms tumor 1-associating protein (WTAP), YTH domain-containing family protein 2 (YTHDF2), fat mass and obesity-associated protein FTO), and AlkB homology 5 (ALKBH5; Yang et al., 2018; Liu et al., 2021). METTL-3 and -14, and WTAP primarily mediate the conversion of adenosine to m^6^A, while demethylases FTO and ALKBH5 can reverse this modification (Widagdo and Anggono, 2018).

Emerging evidence has reported that m^6^A modification is strongly associated with multiple physiological and pathological processes, such as ischemic stroke, traumatic brain injury (TBI), and peripheral nerve injury (Weng et al., 2018; Chokkalla et al., 2019; Wang et al., 2019; Si et al., 2020). As of late, Wang et al. have reported that m^6^A modification was significantly changed in the early period of TBI in mice by m^6^A modified RNA immunoprecipitation sequencing (m^6^A-RIP-seq) and RNA sequencing (RNA-seq; Wang et al., 2019). In the sciatic nerve lesion model, the m^6^A-tagged transcripts encode many regeneration-associated genes and protein translation machinery components in the adult mouse dorsal root ganglion (DRG; Weng et al., 2018). However, the role of m^6^A in SCI remains to be characterized.

This study systematically profiled RNA m^6^A modification landscape by m^6^A-mRNA and lncRNA Epi-transcriptomic microarray in the mouse SCI model. We found altered m^6^A methylation levels following SCI, leading to the change of m^6^A-tagged transcripts. Furthermore, we screened and found that the decreased METTL3-mediated m^6^A modification may be responsible for the hypo-methylation following SCI. Together, this study suggests that m^6^A modifications are involved in the process of SCI, which may be a promising therapeutic target.

## Results

### The global m^6^A levels are decreased after spinal cord injured in mice

**FIGURE 1:**
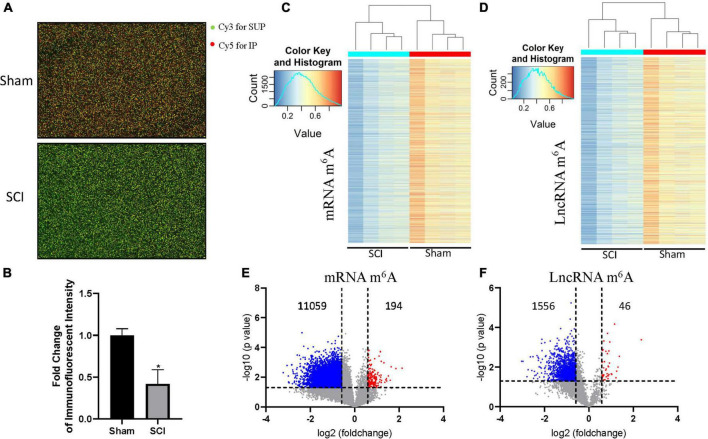
Decrease of the global m^6^A level post-SCI in mice. **(A)** The representative array of images of the SCI group and sham group. Cy3 for Sup and Cy5 for IP. **(B)** Fold change of the immunofluorescent intensity of cy5-labeled immunoprecipitation in the microarray images from SCI group over sham group. **(C)** The heatmap of mRNA m^6^Amethylation level in SCI group and sham group. **(D)** The heatmap of LncRNA m^6^A methylation level in SCI group and sham group. **(E,F)** The volcano plot of mRNA methylation level (194 hyper- and 11,059 hypo-methylation) **(E)** and LncRNA methylation level (46 hyper- and 1,556 hypo-methylation) **(F)** in the SCI group over the sham group. The threshold lines are set at |fold change| ≥ 1.5 and *p* < 0.05 between SCI and sham. *n* = 4/group.

To investigate the role of m^6^A modification in SCI, we established the mouse model with SCI and extracted spinal cord tissues 3 days after surgery. The levels of m^6^A modifications were evaluated by methylated RNA immunoprecipitation and transcriptional microarray analysis. The global m^6^A levels in the SCI group were significantly decreased compared with the sham group, as revealed by the immunofluorescent intensity of cy5-labeled immunoprecipitation in the microarray images (Figures 1A,B). Consistent with the global m^6^A analysis, the levels of transcript-specific m^6^A modification in mRNAs and lncRNAs were also significantly lower in the SCI group than in the sham group (Figures 1B–D). The microarray profiling showed that the m^6^A levels were significantly decreased in 98% mRNAs (194 hyper- and 11,059 hypo-methylation; Figure 1E and Supplementary Table 1) and 97% lncRNAs (46 hyper- and 1,556 hypo-methylation) in the SCI group compared with the sham group (Figures 1D–F and Supplementary Table 2). Together, these data indicated that the global m^6^A methylation levels decreased in the SCI group compared with the sham group, especially the m^6^A enrichment of mRNAs and LncRNAs.

### Gene ontology and KEGG enrichment analyses of differential m^6^A-modified transcripts

**FIGURE 2:**
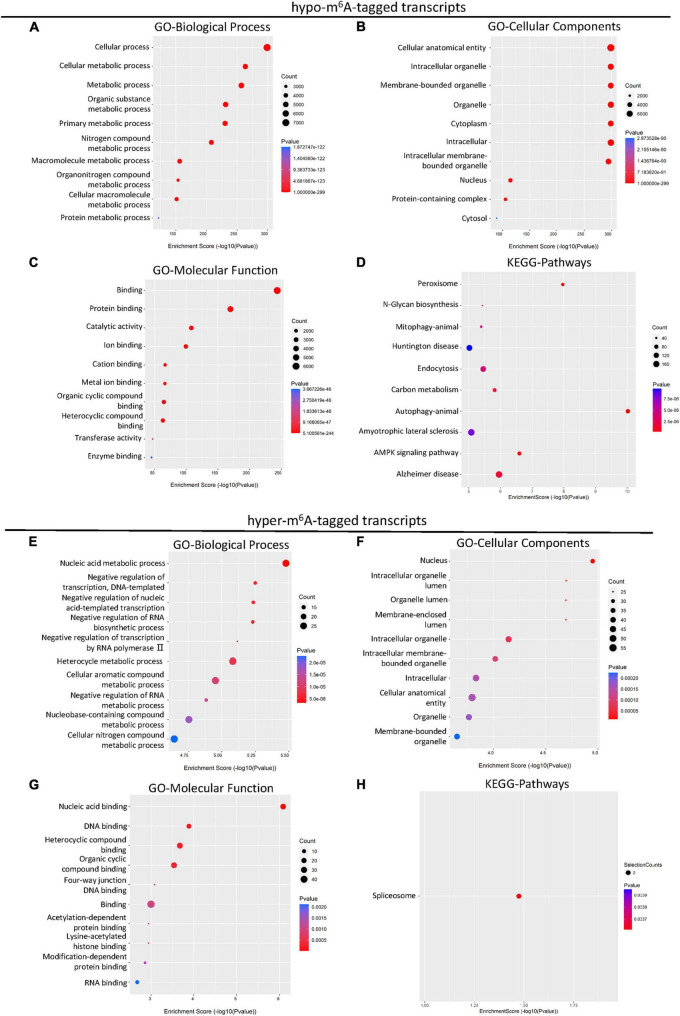
KEGG and GO enrichment analysis of differential m^6^A-tagged transcripts after SCI. **(A–C)** Gene ontology (GO) analysis of hypo-m^6^A-tagged transcripts for biological process (BP), cellular components (CC), molecular function (MF) in the SCI group over the sham group. **(D)** KEGG pathway analysis of hypo-m^6^A-tagged transcripts in SCI group over sham group. **(E–G)** Gene ontology (GO) analysis of hyper-m^6^A-tagged transcripts for biological process (BP), cellular components (CC), molecular function (MF) in SCI group over sham group. **(H)** KEGG pathway analysis of hyper-m^6^A-tagged transcripts in SCI group over sham group.

It has been reported that m^6^A methylation played an essential role in the regulation of mRNA translation in the CNS system (Merkurjev et al., 2018). To further demonstrate the potential function of differential m^6^A-tagged transcripts (mRNAs) after SCI, gene ontology (GO) analysis, and kyoto encyclopedia of genes and genomes (KEGG) analysis were conducted. The results of GO analysis indicated that the hypo-m^6^A-tagged transcripts after the SCI were mainly enriched in the biological process (BP) of the cellular and metabolic processes (Figure 2A). These hypo-m^6^A-tagged transcripts were enriched in the cellular anatomical entity, organelle, and cytoplasm revealed by cellular components (CCs) analysis (Figure 2B). In addition, the molecular functions (MF) of the hypo-m^6^A-tagged transcripts were highly enriched in binding, catalytic activity, and transferase activity (Figure 2C). Moreover, KEGG enrichment was analyzed. The mRNAs with hypo-m^6^A modification after SCI were primarily involved in several pathways namely peroxisome, N-Glycan biosynthesis, mitophagy, endocytosis, carbon metabolism, autophagy, amyotrophic lateral sclerosis, and AMPK signaling (Figure 2D).

Different from hypo-m^6^A-tagged mRNA, hyper-m^6^A-tagged mRNAs were predominantly enriched in BP of nucleic acid metabolism, negative regulation of transcription, and negative regulation of biosynthetic process after the SCI in the GO analysis (Figure 2E). Cellular components analysis demonstrated that hyper-m^6^A-tagged transcripts were mainly enriched in the nucleus, organelle, and its lumens (Figure 2F). The MF enrichments were primarily found in binding terms (Figure 2G). In addition, the KEGG analysis demonstrated that only the spliceosome-related pathway was significantly associated with the hyper-m^6^A-tagged mRNAs (Figure 2H).

### Gene ontology and KEGG enrichment analyses of differential m^6^A-tagged transcripts with altered transcription levels

**FIGURE 3:**
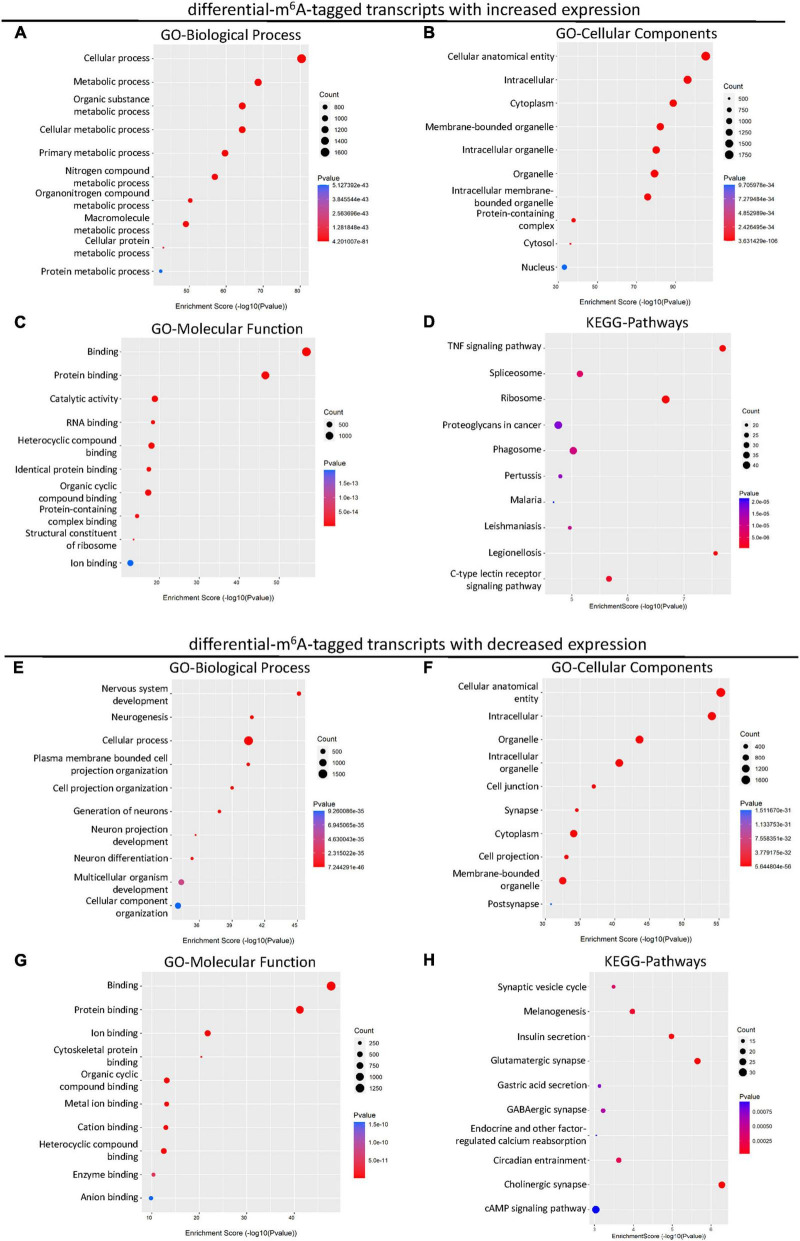
KEGG and GO enrichment analysis of the altered transcripts modified by differential m^6^A after SCI. **(A–C)** Gene ontology (GO) analysis of differential m^6^A-tagged transcripts with increased transcription levels for biological process (BP), cellular components (CC), molecular function (MF) in SCI group over sham group. **(D)** KEGG pathway analysis of differential m^6^A-tagged transcripts with increased transcription levels in SCI group over sham group. **(E–G)** Gene ontology (GO) analysis of differential m^6^A-tagged transcripts with decreased transcription levels for biological process (BP), cellular components (CC), molecular function (MF) in SCI group over sham group. **(H)** KEGG pathway analysis of differential m^6^A-tagged transcripts with decreased transcription levels in SCI group over sham group.

**FIGURE 4:**
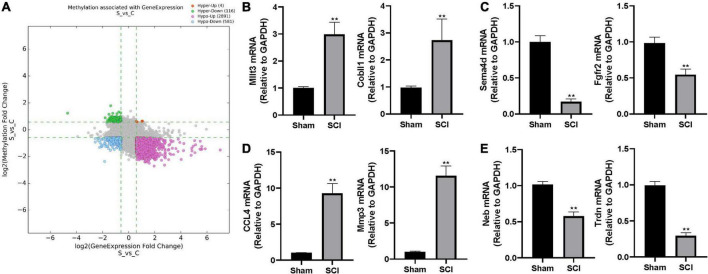
The altered m^6^A level associated with changed gene expression. **(A)** The scatter plot of altered m^6^A level associated with changed gene expression. The threshold lines are set at |fold change| ≥ 1.5 between SCI and sham. Hyper: hypermethylation, Hypo: hypomethylation, up: increased gene expression, down: decreased gene expression. **(B–E)** Validation of randomly selected two genes in different groups by real-time quantitative polymerase chain reaction (qRT-PCR) after SCI. **(B)** hyper-up group, **(C)** hyper-down group, **(**D**)** hypo-up group, **(E)** hypo-down group. Bars indicate mean ± SD; *n* = 5 per group. **p < 0.01 compared with sham group.

Considering the changed m^6^A level of transcripts may not lead to the differences in gene expression, we next explored the differentially expressed genes with altered m^6^A modification after SCI. A total of 2,895 up-regulated and 697 down-regulated mRNA with m^6^A methylation were identified after the SCI (Figure 4A). Then, to reveal the potential role of differentially expressed mRNA, GO and KEGG enrichment analyses were conducted. The GO analysis showed that the up-regulated genes were primarily involved in the cellular and metabolic processes (Figure 3A) and enriched in the cellular anatomical entity, intracellular, cytoplasm, and organelle (Figure 3B). The enrichment of MF was found in binding, catalytic activity, and structural constituent of ribosome (Figure 3C). In addition, the KEGG analysis indicated that the up-regulated mRNAs were significantly related to TNF signaling, spliceosome, ribosome, proteoglycans in cancer, phagosome, and C-type lectin receptor signaling pathway (Figure 3D).

In addition, we analyzed down-regulated genes with altered m^6^A modification. The GO analysis showed that these down-regulated mRNAs were primarily enriched in the BP of cellular process, nervous system development, multicellular organism development, and cellular component organization (Figure 3E). They were mainly located in the cellular anatomical entity, intracellular components, organelle, cell junction, synapse, cytoplasm, cell projection, and post synapse (Figure 3F). The enrichment of MF was primarily found in the binding (Figure 3G). The down-regulated mRNAs were significantly involved in pathways namely, synaptic vesicle cycle, melanogenesis, insulin secretion, glutamatergic synapse, gastric acid secretion, GABAergic synapse, endocrine, and other factor-regulated calcium reabsorption, circadian entrainment, cholinergic synapse, and cAMP signaling (Figure 3H).

### Validation of altered transcription levels to differentially m^6^A-modified genes

To further characterize the differentially expressed genes with altered m^6^A level, we categorized the up-/down-regulated transcripts into four groups: m^6^A hypermethylation with up-regulated transcription levels (hyper-up, 4 genes), m^6^A hypermethylation with down-regulated transcription levels (hyper-down, 116 genes), m^6^A hypomethylation with up-regulated transcription levels (hypo-up, 2891 genes), m^6^A hypomethylation with down-regulated transcription levels (hypo-down, 581 genes; Figure 4A and Supplementary Table 3). We randomly selected two genes from each group and performed real-time quantitative polymerase chain reaction (qRT-PCR) to validate the above observations in the epitranscriptomic microarray analysis. Consistent with our findings, mRNA expressions of Mllt3 and Cobll1 in the hyper-up group were significantly increased after the SCI; whereas the mRNA expression levels of Sema4d and Fgfr2 in the hyper-down group were remarkably decreased after the SCI (Figures 4B,C). The qRT-PCR showed that the expression of CCL4 and Mmp3 increased significantly after the SCI in the hypo-up group (Figure 4D), while, the mRNA levels of Neb and Trdn in hypo-down group were notably lower in the Tran SCI group compared with the sham group (Figure 4E).

### Methyltransferase like 3 is down-regulated following spinal cord injured in mice

**FIGURE 5:**
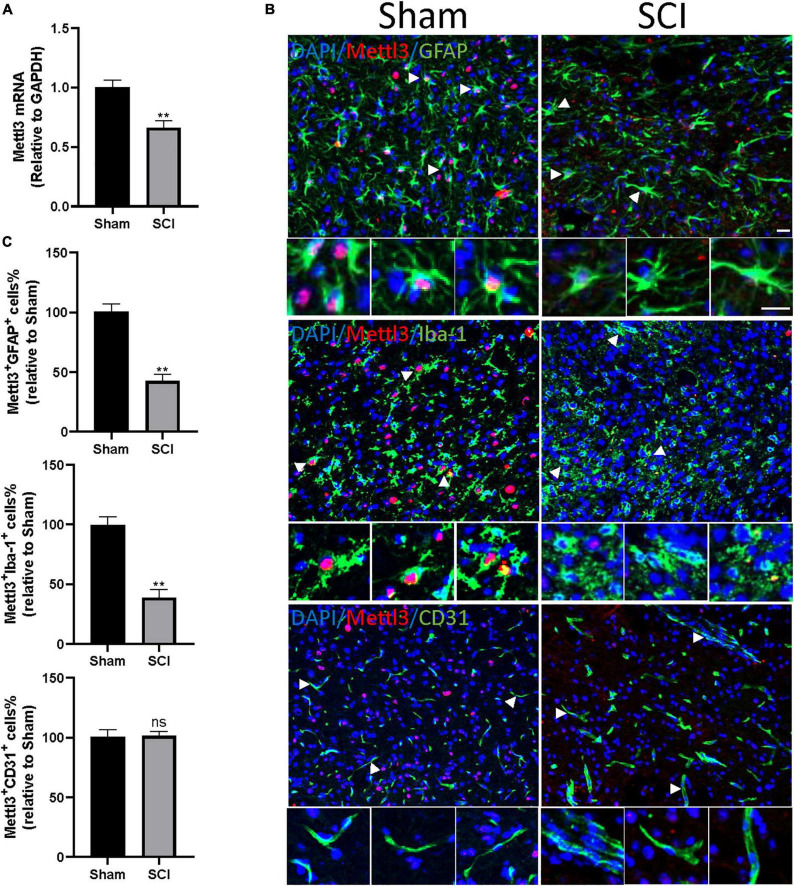
METTL3 is decreased in GFAP^+^ or Iba-1^+^ cells following SCI. **(A)** The decrease of METTL3 mRNA level by qRT-PCR at 3 days after SCI. **(B)** Representative images of spinal cord sections stained for METTL3 (red) with GFAP (green) or Iba-1 (green) or CD31 (green) at 3 days after the SCI and sham group. Scale bar, 50 μm. **(C)** Percentage of number of METTL3^+^GFAP^+^, METTL3^+^Iba-1^+^, and METTL3^+^CD31^+^ in SCI group relative to the sham group. Bars indicate mean ± SD; *n* = 5/group. **p < 0.01 compared with the sham group. ns, no significance.

S-adenosylmethionine (SAM) is a common substrate that functions as a methyl donor for most methyltransferases in important biochemical reactions. Enzyme-linked immunosorbent assay (ELISA) showed that the concentration of SAM did not change significantly after the SCI compared to the sham group (Supplementary Figure 1). To further explore the key regulator in the m^6^Amodification after SCI, the expression of m^6^A methylase complex subunits (METTL3, METTL14, and WTAP) and m^6^A demethylase (FTO and YTHDF2) were screened based on the m^6^A-mRNA and lncRNA Epitranscriptomic Microarraythe RNA-sequence database. A significant decrease in the mRNA expression of METTL3 was observed 3 days after the SCI, consistent with the above observation of the global m^6^A level (Supplementary Table 1). METTL3 is essential to catalyze m^6^A-dependent methylation (Liu et al., 2014). To validate the change of METTL3 in the mouse SCI model, qRT-PCR analysis was conducted. The SCI surgery significantly diminished the expression of METTL3 compared with the sham group (Figure 5A).

We then sought to demonstrate the distribution of METTL3 in different cell types of the spinal cord after trauma. The immunofluorescent staining of METTL3 revealed that the METTL3^+^ cells were positive for astrocyte marker glial fibrillary acidic protein (GFAP) and macrophage/microglia marker allograft inflammatory factor 1 (Iba1), but rarely expressed endothelial marker CD31 (Figure 5B). Interestingly, the numbers of METTL3^+^GFAP^+^ and METTL3^+^Iba-1^+^ cells were significantly lower in the SCI group than in the sham group; while, no significant change in METTL3^+^CD31^+^ cells was observed after the SCI surgery (Figure 5C). In conclusion, these results demonstrate that the change of METTL3 expression after SCI mainly occurred in GFAP^+^ astrocytes, and Iba-1^+^ macrophage/microglia cells.

## Discussion

The SCI is catastrophic trauma of the central nervous system (CNS) that can initiate multiple biological processes and molecule events (Ahuja et al., 2017). As a key mechanism to mediate gene transcription, epigenetics plays a critical role in the response to trauma in the mammalian nervous system (Meng et al., 2017). However, far too few studies have focused on epigenetic changes in SCI (Finelli et al., 2013; Crunkhorn, 2019). Our previous study demonstrated that the epigenetic network is essential for vascular regeneration and functional recovery post-SCI (Ni et al., 2019). The m^6^A methylation plays a significant role in the pathological process of corneal injury, brain injury, and peripheral nerve injury (Weng et al., 2018; Wang et al., 2019; Dai et al., 2021; Zhang et al., 2021). In contrast, various types of cancers and diabetes are associated with lowered m^6^A abundance (Chen et al., 2020; Huang et al., 2021; Lei et al., 2021; Ruan et al., 2021; Wang et al., 2021a; Ye et al., 2021). Therefore, m^6^A homeostasis might be essential for normal physiology, and its disorder leads to pathologies. Nevertheless, the functional relevance of m^6^A modification to post-SCI damage is not yet clear. In the present study, we first demonstrate the m^6^A landscape in the SCI model of mice. The methylated RNA immunoprecipitation combined with microarray analysis showed that the global RNA m^6^A levels were decreased following the SCI. These results indicate that the changed m^6^A modification may involve the pathologies of tissue damage in SCI.

Then, we performed profiling of m^6^A-tagged mRNA and LncRNA after the SCI in mice. Consistent with the decreased global m^6^Amethylation level, 11253 m^6^A peaks were differentially expressed in mRNA transcriptomes after SCI, namely 194 up-regulated and 11,059 down-regulated. In a similar manner, 46 m^6^A peaks were elevated and 1,556 m^6^A peaks were decreased in LncRNA transcriptomes after the SCI. Interestingly, most differential m^6^A-tagged mRNA transcriptomes were hypomethylated, but their transcriptional levels were up-regulated after SCI. This indicates that the level of m^6^A methylation negatively correlated with the transcriptional levels after SCI. Consistently, several previous studies reported that m^6^A mainly functioned in mRNA degradation (Dominissini et al., 2012; Meyer et al., 2012; Wang et al., 2014).

We found that the BP of differential m^6^A-tagged transcripts post SCI were enriched in the cellular and metabolic processes, implying that m^6^A modification may contribute to metabolic alteration after SCI. In general, SCI results in transient or persistent spinal cord metabolic disorder because of post-traumatic ischemia, inflammation, and other mechanisms (Fan et al., 2018), representing m^6^A modification as a potential therapeutic target in the metabolic process after SCI for further study. Cellular Components (CC) of GO analysis suggested that these transcripts were enriched in the cellular anatomical entity, cytoplasm, nucleus, and intracellular organelle, indicating genes modified by m^6^A were widespread in cells following the SCI. In addition, the MF of these hypo- and hyper-methylated genes are enriched in binding, protein binding, and nucleic acid binding, consistent with the broad and critical roles of RNA methylation in gene expression regulation. The KEGG analysis showed that the m^6^A-tagged transcripts were enriched in several pathways, namely spliceosome, peroxisome, autophagy, mitophagy, endocytosis, carbon metabolism, amyotrophic lateral sclerosis, and AMPK signaling, indicating the m^6^A modification may participate in the processes of oxidative stress, autophagy, metabolism, and nervous systems diseases.

Considering the changed m^6^A level of transcripts may not lead to the differences in gene expression, we next conducted the GO and KEGG analyses of the differentially expressed genes with altered m^6^A modification after SCI. Similar to the BP of differential m^6^A-tagged transcripts, the up-regulated transcripts were enriched in the cellular process, cellular metabolic process, and metabolic process. However, noticeably, the down-regulated transcripts were enriched in nervous system development, neurogenesis, and cellular process, indicating the decay of tagged neurodevelopment-and neurogenesis-related transcripts after the SCI. The KEGG analysis showed that the up-regulated transcripts were enriched in the TNF signaling pathway, ribosome, legionellosis, C-type lectin receptor signaling pathway, phagosome, and spliceosome, suggesting m^6^A modification promotes inflammation-related transcripts following the SCI. However, the down-regulated transcripts were enriched in the cholinergic synapse, glutamatergic synapse, insulin secretion, melanogenesis, synaptic vesicle cycle, and circadian entrainment. This indicates that m^6^A-tagged transcripts are involved in synaptic growth, synaptic assembly, and metabolism, thus influencing the communication between axons post-SCI.

The METTL3, METTL14, and WTAP mainly regulate the methylation process of m^6^A, while the FTO and ALKBH5 can reverse this modification (Widagdo and Anggono, 2018; Yang et al., 2018; Liu et al., 2021). Our results showed that only one transcript’s mRNA level of METTL3 significantly decreased post-SCI among these key enzymes in regulating m^6^A modification (Supplementary Table 4). The subsequent qRT-PCR and immunofluorescence data verified the change of METTL3 in mRNA expression and protein expression post-SCI. The mRNA level of another three transcripts of METTL3 did not change significantly, which means that they might not be involved in the change of METTL3 post-SCI. Recent increasing evidence suggests that METTL3, a key RNA N6-adenosine methyltransferase, is involved in the regulation of the nervous system (Hess et al., 2013; Shi et al., 2018; Wang et al., 2018). The METTL3 is abundantly enriched neurogenesis during the early stage (Yoon et al., 2017). Furthermore, conditional METTL3 knockout (cKO) in mice impairs the differentiation of embryonic neural progenitor cells, prolongs cell cycle progression of radial glia, and extends cortical neurogenesis into postnatal stages (Yoon et al., 2017). In addition, silencing METTL3 could significantly promote cell proliferation and migration and induce G0/G1 arrest in some cancers (Li et al., 2017; Chen et al., 2018; Visvanathan et al., 2018). The present study revealed that METTL3 was decreased in the early stage after SCI and predominantly localized and down-regulated explicitly in the GFAP^+^ or Iba-1^+^ cells. Function tests are required to elucidate the effects of the decreased METTL3 on astrocytes or macrophage/microglia after SCI.

Noticeably, the expression of FTO also decreased after the SCI. However, as the RNA demethylase, the change of FTO was inversely correlated with the altered m^6^A level (Weng et al., 2018; Mathiyalagan et al., 2019). Hence, the change of METTL3 was consistent with the decreased trend of global m^6^A after the SCI.

In conclusion, we find that both the level of global m^6^A and the expression of METTL3 are significantly decreased in the mouse SCI model. Profiling of m^6^A-tagged transcripts and subsequent bioinformatics analysis reveal the potential functions of altered m^6^A modified transcripts. Our study suggests that m^6^A modifications could be a potential therapeutic target for SCI.

## Materials and methods

### Establishment of the contusion spinal cord injured model

All the experimental animal protocols were approved by the Ethics Committee of The First Affiliated Hospital of Zhengzhou University for Scientific Research. The animals were kept in specific pathogen-free (SPF) conditions in the Department of Laboratory Animals. The animals were housed in identical environments (temperature 22–24°C; humidity 60–80%) on a 12-h light–dark cycle and fed standard rodent chow *ad libitum* with free unlimited food and water. The 2-month-old mice were anesthetized with ketamine and xylazine by intraperitoneal (i.p.) injection. After laminectomy at T10, moderate contusion injury of the spinal cord was instigated by a modified Allen’s weight drop mechanical assembly (10 g weight at a vertical height of 20 mm, 10 g x 20 mm). Mice in the sham group were only subjected to laminectomy without contusion. Bladders were physically kneaded twice daily until full voluntary or autonomic voiding. Antibiotic (penicillin sodium; North China Pharmaceutical, Shijiazhuang, China) was administered once daily for 3 days post-surgery.

### m^6^A-mRNA and lncRNA epitranscriptomic microarray

#### m^6^A Immunoprecipitation

The 3 μg total RNA and m^6^A spike-in control mixture was added to 300 μl 1 × IP buffer (50 mM Tris–HCl, pH 7.4, 150 mM NaCl, 0.1% NP40, 40U/μl RNase Inhibitor) containing 2 μg anti-m^6^A rabbit polyclonal antibody (Synaptic Systems). The reaction was incubated with head-over-tail rotation at 4°C for 2 h. Dynabeads™ M-280 Sheep Anti-Rabbit IgG (Invitrogen) suspension was blocked with freshly prepared 0.5% BSA at 4°C for 2 h and resuspended in the total RNA-antibody mixture prepared earlier. Then beads were then washed three times with 1 × IP buffer and twice with wash buffer (50 mM Tris–HCl, pH 7.4, 50 mM NaCl, 0.1% NP40, 40 U/μl RNase Inhibitor). The enriched RNA was eluted with Elution buffer (10 mM Tris–HCl, pH 7.4, 1 mM EDTA, 0.05% SDS, 40U Proteinase K) at 50°C for 1 h. The RNA was extracted by acid phenol–chloroform and ethanol precipitated. The immunoprecipitated “IP” fraction contained enriched m^6^A methylated RNAs, and the supernatant “Sup” fraction contained unmodified RNAs.

#### Labeling and hybridization

The “IP” RNAs and “Sup” RNAs were added with equal amounts of calibration spike-in control RNA, amplified as cRNAs, and labeled with Cy3 (green for “Sup”) and Cy5 (red for “IP”) separately using Arraystar Super RNA Labeling Kit (Arraystar). The synthesized cRNAs were further purified by RNeasy Mini Kit (QIAGEN). Then Cy3 and Cy5 labeled cRNAs were combined together and were fragmented. Then, 50 μl hybridization solution was dispensed into the gasket slide and assembled to the mouse m^6^A-mRNA and lncRNA Epitranscriptomic Microarray Arrays (8 × 60 K, Arraystar, Rockville, MD, United States at 65°C for 17 h in an Agilent Hybridization Oven. The hybridized arrays were washed, fixed, and scanned in two-color channels using an Agilent Scanner G2505C. Agilent Feature Extraction software (version 11.0.1.1) was used to analyze acquired array images. Raw intensities of “IP” and “Sup” were normalized with an average of log2-scaled spike-in RNA intensities. The “m^6^A methylation level” was calculated for the percentage of modification based on the “IP” and “Sup” normalized intensities.

#### Bioinformatics analysis

The hierarchical clustering heatmap analysis was performed using the heatmap.2 function in the gplots R package. The heatmaps of differentially m^6^A-methylated lncRNAs and mRNAs were generated and clustered based on the Euclidean distance matric. The present clustergrams represent each transcripts’ row of data across each of the columns of variables as a color block, using stronger intensities of blue color to represent lower levels of the m^6^A methylation, and increasing intensities of red color to represent higher levels. The volcano plot analysis was performed using the ggplot2. function in the gplots R package. The GO analysis was performed using the topGO package in the R environment for statistical computing and graphics, and Pathway analysis was calculated by Fisher’s exact test.

### Immunohistochemistry

The spinal cord sections were washed in PBS for 15 min, then with 1% PBST (1% Triton X-100 in PBS) for 30 min two times. The slices were incubated with a blocking solution (5% BSA in 1% PBST) at room temperature for 1 h. Primary antibodies, namely, anti-METTL3 (Abcam, ab195352, and 1:500), anti-CD31 (R&D Systems, Inc., FAB3628G-100, and 1:200), anti-GFAP (Abcam, ab53554, and 1:500), anti-Iba-1 (Wako, 01127991, and 1:800) were incubated at 4°C overnight. The corresponding secondary antibodies (Abcam and 1:500) were incubated for 1 h at room temperature. There were five samples in the Sham and SCI group, respectively. For each sample, we selected five slices, and five fields of view for each slice under 200⋅magnification. The range of each field of view is 600 × 500 μm, and there are 915 ± 85.5 cells on an average in each field of view. In total, 2.5⋅10^4^ cells were used for each sample for cell count.

### Ribonucleic acid isolation and qRT-PCR

The spinal cord tissue for RNA isolation is 1 cm in length, around the injury site. According to the manufacturer’s protocol, total RNA was extracted using TRIzol (Invitrogen). The qRT-PCR was performed using the PrimeScript RT reagent Kit (Takara) and SYBR Premix Ex Taq (Takara) following the specifications. For the quantification of mRNA expression, primers were provided by Sangon Biotech (Shanghai, China). The expression of GAPDH was used as an internal control. The analysis of gene expression was performed using the 2 ^–ΔΔCt^ method.

### Enzyme-linked immunosorbent assay

The spinal cord tissues were collected from the SCI group and the Sham group. The tissue supernatant was prepared and the concentration of SAM was determined by enzyme-linked immunosorbent assay (ELISA; Cloud-Clone Corp., Wuhan, China) according to the manufacturer’s instructions (*n* = 4 per group), respectively.

### Statistical analysis

All the data were presented in the form of means ± SD. The *t*-test was used to compare the differences between the groups. All the statistical analyses were carried out using SPSS 19.0 software. *p* < 0.05 was considered statistically significant.

## Data availability statement

The original contributions presented in this study are included in the article/Supplementary material, further inquiries can be directed to the corresponding authors.

## Ethics statement

This animal study was reviewed and approved by Ethics Review Committee in Life Science of Zhengzhou University.

## Author contributions

SN: data curation, formal analysis, and writing – original draft. ZL: writing – original draft, methodology, and data curation. YF: project administration and methodology. WZ: validation and investigation. WP: writing – review and editing and supervision. HZ: funding acquisition and writing – review and editing. All authors contributed to the article and approved the submitted version.
