# Supplementary material for: Alteration of m6A epitranscriptomic tagging of ribonucleic acids after spinal cord injury in mice
Source: Front Neurosci. 2022 Aug 25;16:904573. doi: 10.3389/fnins.2022.904573 (PMC9454195; doi:10.3389/fnins.2022.904573)
Supplement: Supplementary file 4 [file Table_4.docx]

**Supplementary Tables**

Table 4. The expression of m^6^A methylase complex subunits (METTL3 METTL14, and WTAP) and m^6^A demethylase (FTO, YTHDF2) based on the RNA-sequence database.





*p≤0.05, ^#^fold change consistent with the global m6a level after SCI
