# Supplementary material for: Alteration of m6A epitranscriptomic tagging of ribonucleic acids after spinal cord injury in mice
Source: Front Neurosci. 2022 Aug 25;16:904573. doi: 10.3389/fnins.2022.904573 (PMC9454195; doi:10.3389/fnins.2022.904573)
Supplement: Supplementary file 5 [file Image_1.pdf]

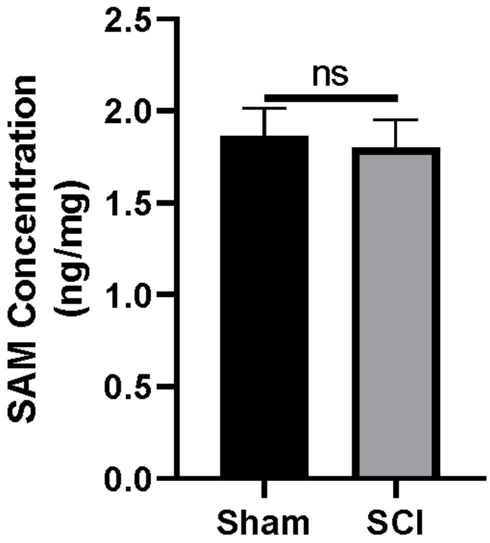

Supplementary Figure 1 The concentration of SAM does not change significantly at 3 days after SCI by ELISA. Bar indicates mean ± SD; n = 4/group. ns, no significance, compared with sham group.
